# Impact of *Candida albicans NDT80* and *UME6* on biofilm formation and fluconazole susceptibility

**DOI:** 10.1128/msphere.00014-26

**Published:** 2026-03-27

**Authors:** Katharina Goerlich, Aaron P. Mitchell

**Affiliations:** 1 Department of Microbiology, University of Georgia 189270, Athens, Georgia, USA; University of Guelph, Guelph, Ontario, Canada

**Keywords:** *Candida albicans*, biofilm formation, filamentation, transcriptional regulation, fluconazole susceptibility

## Abstract

The microbiome-associated fungus *Candida albicans* is an opportunistic pathogen. Virulence traits include its ability to produce biofilm, a surface-associated growth form that persists on mucosae and implanted medical devices. *C. albicans* clinical isolates vary in ability to produce biofilm and the constituent filamentous cell types. Here, we focus on two transcription factors that promote filamentation and biofilm formation, Ndt80 and Ume6. We address two questions. First, how variable is the impact of Ndt80 among *C. albicans* strains? Second, what is the genetic interaction between *NDT80* and *UME6*? We find that Ndt80 is required for filamentation and biofilm formation in five clinical isolates in addition to the reference strain SC5314, where Ndt80 function has been well established. RNA-sequencing (RNA-seq) data indicate that *UME6* RNA levels are reduced in an *ndt80*Δ/Δ mutant, possibly a result of altered *RME1* and *WOR1* expression, both of which control *UME6*. Increased expression of *UME6* in *ndt80*Δ/Δ mutants of three strain backgrounds restores filamentation and biofilm formation, though RNA-seq assays indicate that it does not suppress the overall *ndt80*Δ/Δ gene expression defect. Ndt80 has an additional role in promoting tolerance to the antifungal drug fluconazole, an inhibitor of ergosterol synthesis. This *ndt80*Δ/Δ phenotype varies considerably among clinical isolates. In three strains tested, increased expression of *UME6* in *ndt80*Δ/Δ mutants enhances their susceptibility to fluconazole. Therefore, our results show an unexpected relationship between Ume6 expression and azole drug sensitivity. To our knowledge, Ume6 has previously been understood to function only in filamentation, biofilm formation, and related processes.

**IMPORTANCE:**

Our focus is the fungal pathogen *Candida albicans*. Two traits, biofilm/hypha formation and azole resistance, are major drivers of its infection ability. We examine the roles of two biofilm transcriptional regulators, Ndt80 and Ume6, in several *C. albicans* clinical isolates. Prior studies in one strain background (SC5314) indicated that Ndt80 controls both biofilm/hypha formation and azole drug susceptibility and that Ume6 controls biofilm/hypha formation. The four new findings here are that (i) Ndt80 effects on fluconazole sensitivity vary considerably with strain background; (ii) Ndt80 is required for filamentation and biofilm formation in multiple clinical isolates; (iii) the Ndt80 target Ume6 contributes to Ndt80 control of filamentation and biofilm formation in multiple clinical isolates; and (iv) Ume6 influences fluconazole vulnerability, the first Ume6 function to our knowledge that is unrelated to filamentation.

## INTRODUCTION

*Candida albicans* is a natural fungal constituent of the human mucosal flora (1, 2). It is also an opportunistic pathogen that infects a diverse patient population (2–4). Risk factors include immune system deficiencies and presence of an implanted medical device. *C. albicans* is a significant pathogen worldwide, and the World Health Organization has placed *C. albicans* in its critical priority group of fungal pathogens (5). Major virulence traits include the ability to produce biofilm, a structured surface-associated growth form that is tolerant to host defenses and antifungal drugs (6, 7).

Biofilm formation depends upon the transition from yeast to filamentous cell chains called hyphae and pseudohyphae (1). This transition is regulated by a network of transcription factors (TFs) that govern expression of numerous genes required for biofilm formation and, often, many other genes as well (8). We focus here on two of those TFs, Ndt80 and Ume6. Ndt80 belongs to a large eukaryotic TF family and is required in *C. albicans* for biofilm formation, filamentation, and normal susceptibility to azole antifungal drugs (9–11), which inhibit ergosterol synthesis (12). Ndt80 binds directly to promoters of many genes required for biofilm formation, filamentation, ergosterol biosynthesis, and drug efflux, as well as promoters of other genes that specify biofilm regulatory TFs (10, 11, 13). It has been called a master regulator of biofilm formation (8).

Ume6 is a C_6_Zn_2_ zinc cluster TF (14). It was discovered as a regulator of hyphal elongation that is expressed only under filamentation-inducing conditions (14). Engineered expression of *UME6* bypasses many environmental signals that induce filamentation and enables filamentation and biofilm formation in cells lacking Efg1, another master regulator (15, 16). DNA binding and gene activation by Ume6 depend upon its association with TFs that include Efg1, Ndt80, and Upc2 (17).

In this study, we sought to address two questions. First, how variable is the impact of Ndt80 among *C. albicans* strains? Other biofilm regulators present substantial genotype-phenotype variation (18–22). Second, what is the genetic interaction between *NDT80* and *UME6*? Gene expression data suggest that Ume6 may act downstream of Ndt80 (10, 11), but to our knowledge, that hypothesis has not been tested. Our analysis here reveals an unexpected relationship between Ume6 expression and azole drug sensitivity. To our knowledge, Ume6 has previously been known to affect only filamentation and biofilm formation.

## RESULTS

### Ndt80 and background effects

*C. albicans* Ndt80 is considered a positive regulator of filamentation and biofilm formation based on extensive characterization of *ndt80*Δ/Δ mutants in reference strain SC5314 and derivatives (10, 11, 13). We have found that the impact of filamentation and biofilm regulatory mutations can vary considerably with genetic background (18–22). To assess Ndt80 function across a panel of *C. albicans* strains, we constructed and analyzed *ndt80*Δ/Δ mutants in six different clinical isolates (23). The isolates included SC5314 (clade 1), P76067 (clade 2), P57055 (clade 3), P87 (clade 4), P75063 (clade 4), and P78048 (clade 1). We have used the first four strain backgrounds in several genotype-phenotype studies; we could not obtain *ndt80*Δ/Δ homozygotes in the fifth strain we have often used, P75010. Therefore, P75063 and P78048 were included as additional representatives of two major clades.

**Fig 1:**
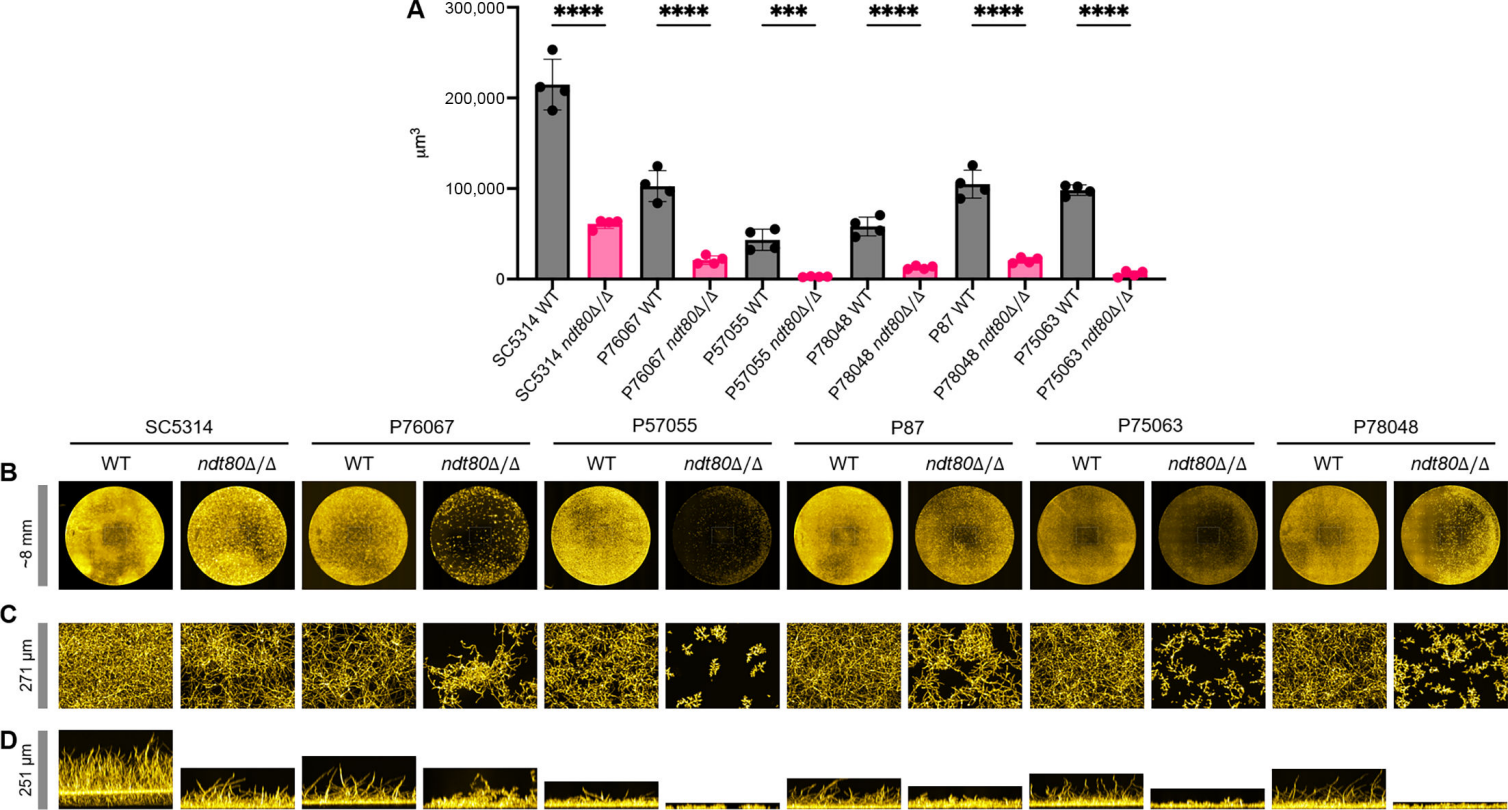
Biofilm assays. *Candida albicans* wild-type (*NDT80*+/+) and *ndt80*Δ/Δ strains were assayed for biofilm formation *in vitro*. Strains were grown in RPMI in a 96-well plate at 37°C for 24 h. Biofilms were fixed and stained with calcofluor white and imaged using a Keyence BZ-X800E fluorescence microscope. Each strain was tested in at least three independent 96-well plate biofilm experiments. (**A**) Biofilm volume measurements were obtained from four independent wells in 96-well plate assays. (**B**) Representative biofilm 96-well plate apical navigation views. (**C**) Representative biofilm 96-well plate apical projection views. (**D**) Representative biofilm 96-well plate side projection views, generated from the same location used in row C. Scale bars (at the left of each row): (**B**) ~8 mm; (**C**) 271 µm; (**D**) 251 µm. A one-way analysis of variance (Brown-Forsythe test) test was used to determine statistical significance, as indicated by asterisks: *, ≤0.05; **, ≤0.01; ***, ≤0.001; ****, ≤0.0001.

To assess the role of Ndt80 in biofilm formation, wild-type and *ndt80*Δ/Δ strains were incubated in RPMI medium at 37°C for 24 h in a 96-well plate biofilm format. Biofilm volume measurements indicated that the wild-type strains varied considerably in biofilm formation ability (Fig. 1A), as expected from our previous studies of these strains in RPMI + serum (18–20). These measurements showed that *ndt80*Δ/Δ mutants in each background had significant defects in biofilm formation (Fig. 1A). Navigation views, which are apical images of biofilm over the entire sample well, indicated that *ndt80*Δ/Δ mutant biofilms were more sparse than the corresponding wild-type biofilms (Fig. 1B). The mutant biofilms also had more prominent clusters of cells than the wild-type biofilms. In the P57055, P75063, and P78048 backgrounds, representative clusters had some elongated cells and pseudohyphae, but few if any hyphae (Fig. 1C). Side view images indicated that several mutant biofilms had less depth than the corresponding wild-type biofilms (Fig. 1D). We conclude that *NDT80* has a positive role in biofilm formation in multiple clinical isolates. In addition, the role of *NDT80* is complex in that it affects biofilm cell morphology as well as biofilm biomass.

**Fig 2:**
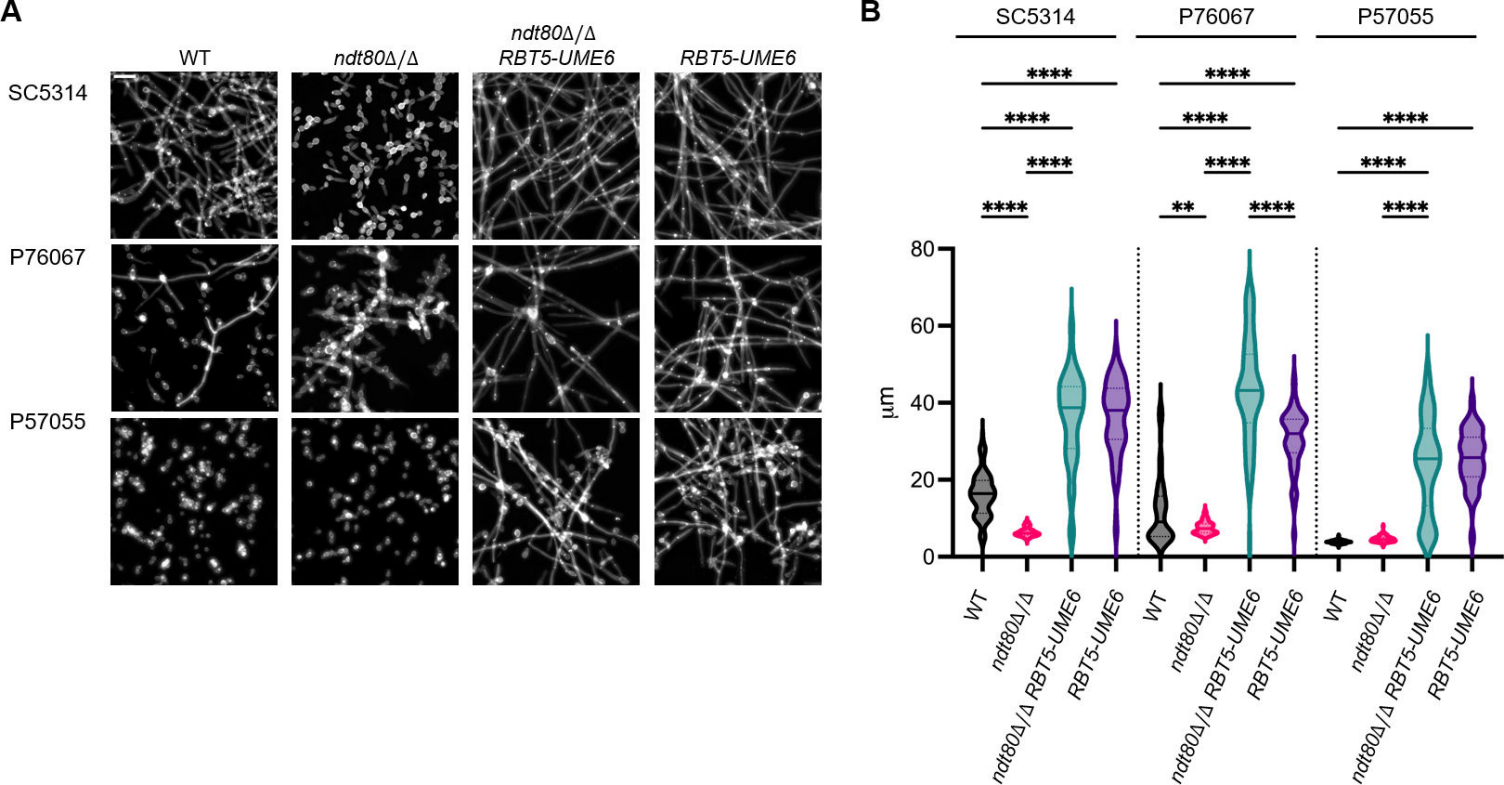
Filamentation assays. Cells were grown overnight in yeast extract peptone dextrose + 50 µM bathophenanthrolinedisulfonate (BPS), then inoculated into prewarmed RPMI for 4 h at 37°C, and treated with proteinase K and stained with calcofluor white. Addition of the iron chelator BPS was necessary to activate *RBT5-UME6* expression. (**A**) Representative fields of view. The scale bar (in the SC5314 wild type [WT] image) is 20 µm. (**B**) Cell length measurements. Cell length was measured for the strains in panel A. At least three fields of view were measured, and 100 cells or all cells were measured in each field. A one-way analysis of variance (Brown-Forsythe test) test was used to determine statistical significance, as indicated by asterisks: *, ≤0.05; **, ≤0.01; ***, ≤0.001; ****, ≤0.0001.

To assess the role of Ndt80 in filamentation, wild-type and *ndt80*Δ/Δ strains were incubated in RPMI at 37°C for 4 h and stained with calcofluor white, and then cell morphology was visualized and quantified (Fig. 2). We focused on three strain backgrounds: SC5314, P76067, and P57055. In the SC5314 background, the *ndt80*Δ/Δ mutant was defective in filamentation, as indicated by visual inspection (Fig. 2A) and cell length measurements (Fig. 2B). In the P76067 background, the *ndt80*Δ/Δ mutant produced aberrant filaments with a seemingly swollen appearance (Fig. 2A) and diminished cell length (Fig. 2B). In the P57055 background, neither the wild type nor the *ndt80*Δ/Δ mutant underwent filamentation under these conditions (Fig. 2A and B). After a 24-h incubation in RPMI, the P57055 wild type underwent filamentation, and the P57055 *ndt80*Δ/Δ mutant was qualitatively defective (Fig. S1). Similar results were obtained for the P87, P75063, and P78048 backgrounds (Fig. S1). The aberrant morphology of *ndt80*Δ/Δ mutants observed here extends the observations of Sellam et al. (10) and Min et al. (9) to multiple genetic backgrounds. We conclude that *NDT80* has a positive role in filamentation in multiple clinical isolates.

**Fig 3:**
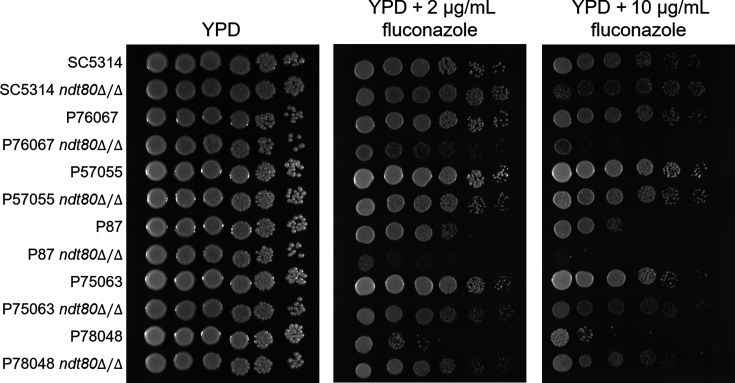
Fluconazole susceptibility assays. Wild-type strains and *ndt80*∆/∆ mutants in the indicated strain backgrounds were assayed for fluconazole sensitivity. Strains were grown overnight in yeast extract peptone dextrose (YPD) at 30°C. Strains were then diluted to an OD_600_ ~3 and spotted on YPD, YPD + 2 µg/mL fluconazole, and YPD + 10 µg/mL fluconazole plates. Plates were then incubated at 30°C for 48 h and imaged. Representative images are shown; each spot plate assay was performed in three independent experiments.

Ndt80 is also required for normal azole drug susceptibility in reference strain SC5314 derivatives (11, 24). We assessed this phenotype with spotting assays of wild-type and *ndt80*∆/∆ strains on yeast extract peptone dextrose (YPD), YPD + 2 µg/mL fluconazole, and YPD + 10 µg/mL fluconazole plates at 30°C (Fig. 3). The strains grew similarly on YPD, and the wild-type strains varied in their sensitivity to fluconazole. In the SC5314, P76067, and P87 backgrounds, *ndt80*Δ/Δ mutants displayed greater fluconazole sensitivity than the wild-type strain. In the P57055 and P75063 backgrounds, the *ndt80*Δ/Δ mutants displayed similar fluconazole sensitivity to the wild type. In the P78048 background, the *ndt80*Δ/Δ mutant was more resistant to fluconazole than the wild type. This unexpected phenotypic behavior was observed with five additional independent *ndt80*Δ/Δ mutant isolates in the P78048 background (Fig. S2). We conclude that the impact of Ndt80 on fluconazole sensitivity is complex; the *ndt80*Δ/Δ mutant phenotype in this regard is among the most variable among strains that we have observed.

### Gene expression impact of *ndt80*Δ/Δ

To gain insight into the basis for phenotypes associated with *ndt80*Δ/Δ, we conducted RNA-sequencing (RNA-seq). Cells were grown under strong hypha-inducing conditions (RPMI at 37°C for 4 h with vigorous shaking). We used wild-type and *ndt80*Δ/Δ strains in the SC5314 background. Differential gene expression was calculated for the mutant compared to the wild-type strain, using conventional cutoffs of log_2_ fold change (LFC) >1 or <−1, and an adjusted *P*-value of <0.05.

**Fig 4:**
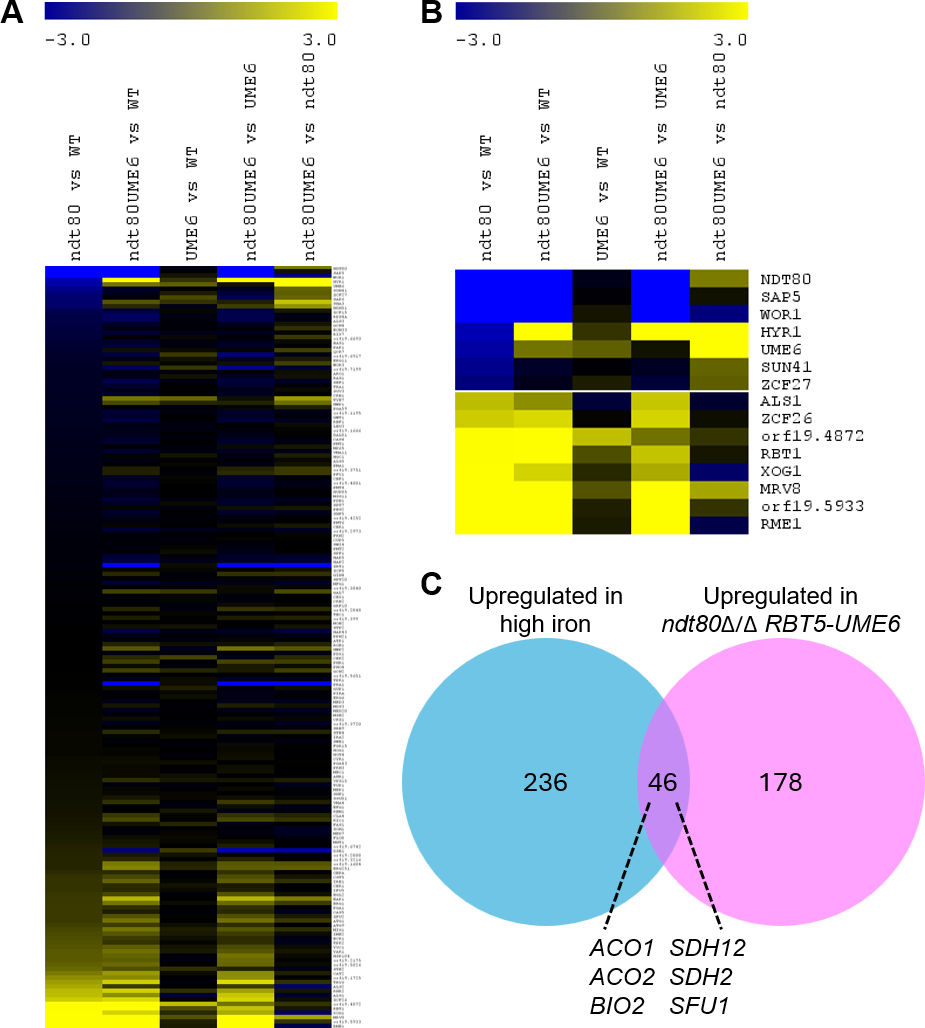
Gene expression changes. (**A and B**) Heat maps present the gene expression changes associated with *ndt80*∆/∆, *ndt80*∆/∆ *RBT5-UME6*, and *RBT5-UME6* strains in the SC5314 background. Color saturation represents log_2_ fold change (LFC) values for the comparisons (left to right) *ndt80*Δ/Δ vs wild type, *ndt80*Δ/Δ *RBT5-UME6* vs wild type, *RBT5-UME6* vs wild type, *ndt80*Δ/Δ *RBT5-UME6* vs *RBT5-UME6*, and *ndt80*Δ/Δ *RBT5-UME6* vs *ndt80*Δ/Δ. Blue represents downregulation; yellow represents upregulation; the scale is LFC −3 to LFC +3. (**A**) Expression changes for 174 genes with the phenotype descriptor “biofilm formation: decreased” in the *Candida* Genome Database (25). (**B**) Expression changes for the genes from panel A that are most altered in the comparison of *ndt80*Δ/Δ vs wild type. Complete RNA-sequencing data are in Table S1. (**C**) Overlap between 226 genes upregulated in the *ndt80*Δ/Δ *RBT5-UME6* strain versus the *ndt80*Δ/Δ strain (Table S1) and 282 genes upregulated in strain SC5314 grown in high iron versus low iron media (26).

Comparison of the *ndt80*Δ/Δ and wild-type strains showed that 778 genes were differentially expressed (Table S1). Gene Ontology (GO) enrichment analysis (Table S1) was not informative with regard to the mutant filamentation and biofilm defect. Therefore, we examined specifically the expression of 174 genes (Fig. 4A; Table S1) with the phenotype descriptor “biofilm formation: decreased” in the *Candida* Genome Database (25). We observed that the SC5314 *ndt80*Δ/Δ mutant had elevated expression of *RME1* and decreased expression of *WOR1* and *UME6* (Fig. 4B). Filamentation and biofilm formation are negatively regulated by Rme1 (21) and positively regulated by Ume6 (14, 15) and in some contexts Wor1 (19). Recent studies indicate that Ume6 functions downstream of Rme1 and Wor1 (19, 21). Therefore, we considered the hypothesis that the Rme1-Wor1-Ume6 module mediates the impact of Ndt80.

### Phenotypic impact of *UME6* expression

*UME6* expression is governed by both Rme1 and Wor1 (19, 21). Therefore, to see whether the Rme1-Wor1-Ume6 module may mediate the impact of Ndt80, we determined whether increased *UME6* expression may suppress *ndt80*Δ/Δ mutant phenotypes. To test suppression, we introduced the *RBT5* promoter 5′ of the *UME6* coding region in a panel of *NDT80+/+* and *ndt80*Δ/Δ strains. The *RBT5* promoter is activated by iron limitation (27), which is imposed when filamentation is induced in RPMI medium. The *RBT5-UME6* construct was homozygous in all strains. We used the same strain backgrounds—SC5314, P76067, and P57055—used above to quantify filamentation.

**Fig 5:**
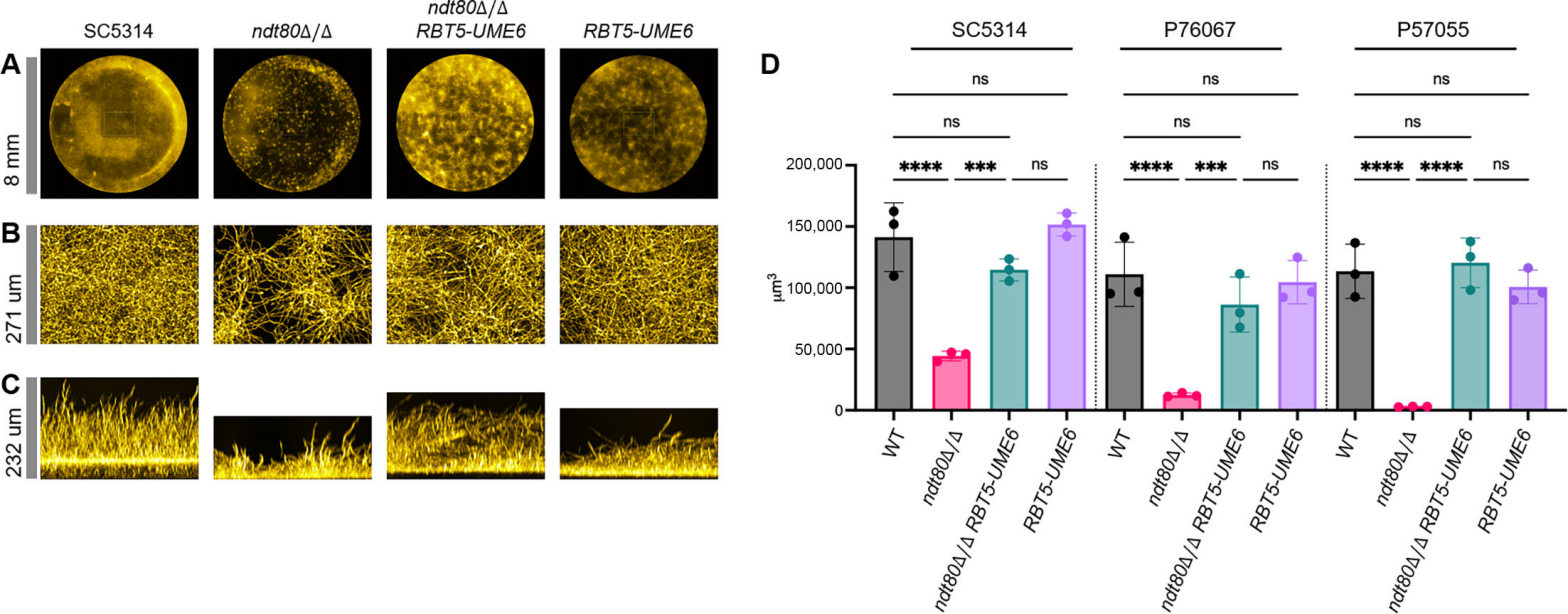
Biofilm assays. Cells were grown overnight in yeast extract peptone dextrose + 50 µM bathophenanthrolinedisulfonate (BPS), then inoculated into prewarmed RPMI in 96-well plates, and grown at 37°C for 24 h. Biofilms were washed and stained with calcofluor white. All strains shown here are from the SC5314 background; strains from the P76067 and P57055 background, along with additional *ndt80*∆/∆ *RBT5-UME6* and *RBT5-UME6* isolates from the SC5314 background, are shown in Fig. S3. (**A**) Representative biofilm 96-well plate apical navigation views. (**B**) Representative biofilm 96-well plate apical projection views. (**C**) Representative biofilm 96-well plate side projection views. Scale bars (at the left of each row): (**B**) ~8 mm; (**C**) 271 µm; (**D**) 232 µm. (**D**) Biofilm volume measurements from three independent 96-well plate assays. A one-way analysis of variance (Brown-Forsythe test) test was used to determine statistical significance, as indicated by asterisks: *, ≤0.05; **, ≤0.01; ***, ≤0.001; ****, ≤0.0001; ns, not significant. Biofilm growth and imaging was performed at least three times independently for each sample.

We first assayed biofilm formation. In all three backgrounds, biofilm formation was severely reduced by the *ndt80*Δ/Δ mutation (Fig. 5; Fig. S3). Presence of *RBT5-UME6* in each *ndt80*Δ/Δ mutant improved biofilm production significantly (Fig. 5D). Apical navigation views of biofilms showed that the patchy distribution of biofilm typical of *ndt80*Δ/Δ mutants was still evident in *ndt80*Δ/Δ *RBT5-UME6* strains (Fig. 5A; Fig. S3). However, a patchy biofilm distribution was also evident in the *RBT5-UME6* strains (Fig. 5A), perhaps a result of the tendency of the strain to filament in preculture conditions. Overall, our data indicate that *UME6* expression largely overcomes the biofilm production defect of *ndt80*Δ/Δ mutants in all three strain backgrounds.

We also assayed filamentation (Fig. 2A and B). Filamentation assays were conducted in RPMI at 37°C for 4 h. The *ndt80*Δ/Δ mutants were defective in filamentation in SC5314 and P76067; wild-type P57055 did not filament under these conditions, nor did the P57055 *ndt80*Δ/Δ mutant. Presence of *RBT5-UME6* in the wild-type strains improved filamentation, as reflected by cell length quantification (Fig. 2B). By this measure, *RBT5-UME6* fully overcame any *ndt80*Δ/Δ filamentation defect in SC5314 and P57055 and improved filamentation dramatically in the P76067 *ndt80*Δ/Δ mutant. We conclude that *RBT5-UME6* is partially or fully epistatic to *ndt80*Δ/Δ with respect to filamentation.

**Fig 6:**
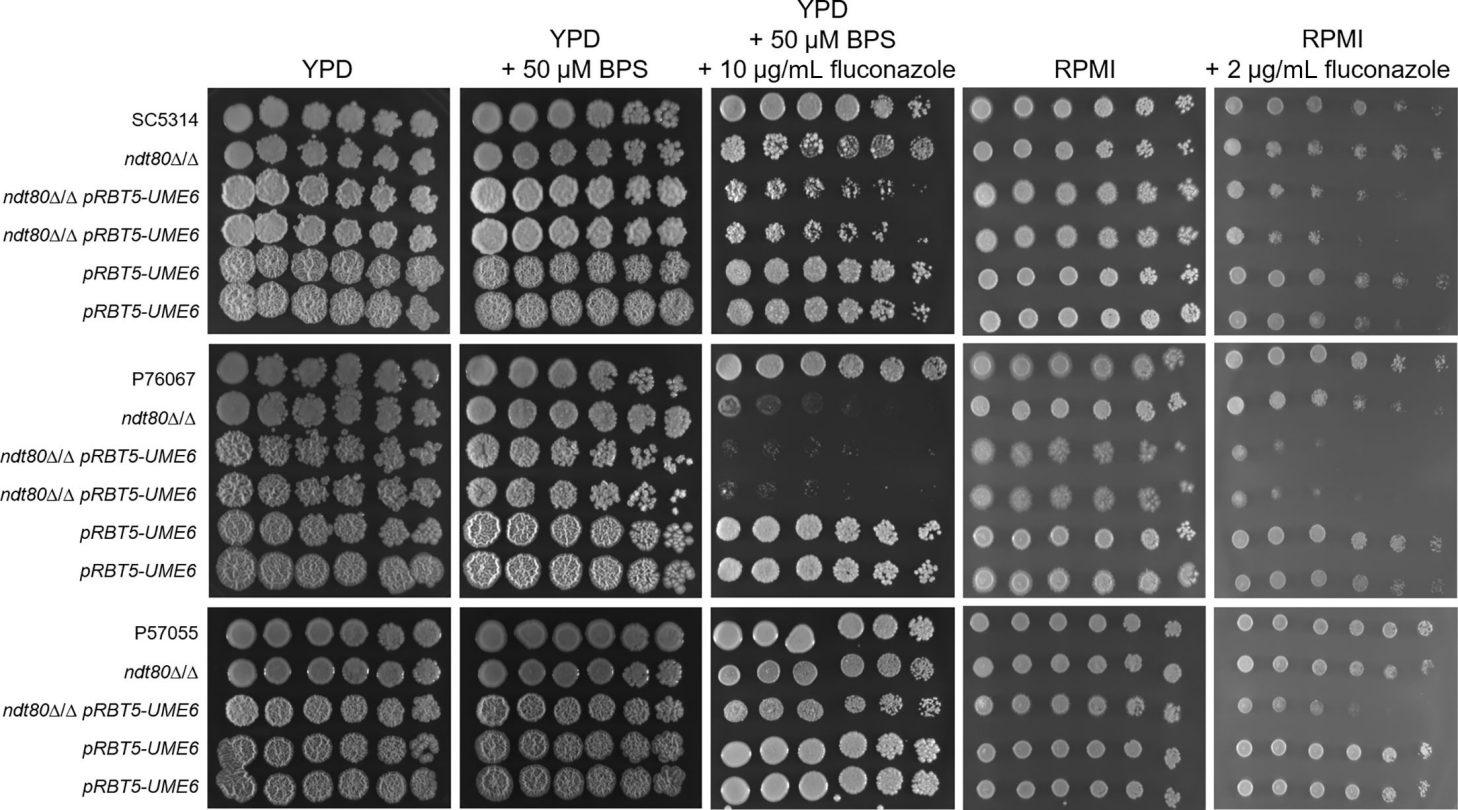
Fluconazole susceptibility assays. Wild type, *ndt80*∆/∆, *ndt80*∆/∆ *RBT5-UME6*, and *RBT5-UME6* isolates in three strain backgrounds were assayed for fluconazole sensitivity. Strains were grown overnight in yeast extract peptone dextrose (YPD) + 50 µM bathophenanthrolinedisulfonate (BPS) at 30°C. Strains were then diluted to an OD_600_ ~3 and were spotted on YPD, YPD + 50 µM BPS, YPD + 50 µM BPS +10 µg/mL fluconazole, RPMI, and RPMI + 2 µg/mL fluconazole plates. Plates were incubated at 30°C for 48 h and imaged. Independent isolates of *ndt80*∆/∆ *RBT5-UME6* and *RBT5-UME6* strains were included if available. Representative images are shown; each spot plate assay was performed at least three times independently.

Finally, we examined the impact of *RBT5-UME6* on fluconazole susceptibility, using spotting assays on YPD + 10 µg/mL fluconazole plates at 30°C (Fig. 6). The plates included a 50 µM bathophenanthrolinedisulfonate (BPS), an iron chelator, to stimulate expression from the *RBT5* promoter (27). In the SC5314 and P76067 backgrounds, the *ndt80*Δ/Δ mutants presented greater fluconazole sensitivity than the wild-type strains, as expected from earlier experiments. Presence of *RBT5-UME6* in these *ndt80*Δ/Δ mutants caused still greater fluconazole sensitivity (Fig. 6). In the P57055 background, the *ndt80*Δ/Δ mutant seemed only marginally more fluconazole sensitive than the wild-type strain, as expected from earlier experiments. Presence of *RBT5-UME6* in this *ndt80*Δ/Δ mutant caused little if any greater fluconazole sensitivity (Fig. 6). We also assayed fluconazole sensitivity of these strains in RPMI medium (Fig. 6), which is low in iron. In RPMI, fluconazole sensitivity of the *ndt80*Δ/Δ mutants was minimal. However, in all three backgrounds, the *ndt80*Δ/Δ *RBT5-UME6* strains presented increased fluconazole sensitivity compared to wild-type, *ndt80*Δ/Δ, and *RBT5-UME6* strains (Fig. 6). Therefore, the combination of *ndt80*Δ/Δ and *RBT5-UME6* alleles leads to fluconazole hypersensitivity in two strain backgrounds in YPD + BPS and in all three strain backgrounds in RPMI.

### Gene expression impact of *RBT5-UME6*

To gain insight into the basis for phenotypes associated with *the RBT5-UME6* mutations, we conducted RNA-seq under the same conditions as for the wild-type and *ndt80*Δ/Δ strains. We included an *RBT5-UME6* strain that was otherwise wild-type and an *ndt80*Δ/Δ *RBT5-UME6* strain, again in the SC5314 background. Differential gene expression was estimated with the conventional cutoffs as above (Table S1).

Comparison of the *ndt80*Δ/Δ *RBT5-UME6* and *ndt80*Δ/Δ strains showed that 310 genes were differentially expressed (Table S1). Among biofilm determinants, the genes *HYR1*, *SHA3*, *NDH51*, *HWP1*, *SAP6*, and *SUN41* were all upregulated in the *ndt80*Δ/Δ *RBT5-UME6* strain (Fig. 4; Table S1) and may contribute to its improved filamentation and biofilm formation. More broadly, the 86 genes downregulated in the *ndt80*Δ/Δ *RBT5-UME6* strain were modestly enriched for stress response and iron transporter genes; the 224 genes upregulated in the *ndt80*Δ/Δ *RBT5-UME6* strain were enriched for functions related to iron-sulfur cluster binding and heme binding (Table S1). In fact, the upregulated group included 46 genes that are also upregulated in high iron versus low iron growth conditions (26), including those specifying the repressor of iron utilization genes Sfu1, and iron-utilizing enzymes aconitase (*ACO1* and *ACO2*), succinate dehydrogenase (*SDH2* and *SDH12*), and biotin synthase (*BIO2*) (Fig. 4C). A simple interpretation is that *RBT5-UME6* expression alters iron homeostasis in the context of the *ndt80*Δ/Δ background.

Studies above indicated that the *RBT5-UME6* mutation by itself causes little phenotypic alteration in RPMI medium. In keeping with the biological observations, comparison of the *RBT5-UME6* and wild-type strains showed that only 14 genes were significantly altered in expression (Table S1; LFC > 1 or < −1, and adjusted *P*-value < 0.05). The impact of *RBT5-UME6* expression is therefore much more modest in a wild-type background than in an *ndt80*Δ/Δ background.

## DISCUSSION

In this study, we investigated the influence of *NDT80* and *UME6* on filamentation, biofilm formation, and fluconazole sensitivity in several clinical isolates of *C. albicans*. Our four new findings are that (i) Ndt80 effects on fluconazole sensitivity vary considerably with strain background; (ii) Ndt80 is required for filamentation and biofilm formation in multiple clinical isolates; (iii) the Ndt80 target Ume6 contributes to Ndt80 control of filamentation and biofilm formation in multiple clinical isolates; and (iv) Ume6 influences fluconazole vulnerability in *ndt80*Δ/Δ mutants of several backgrounds, the first Ume6 function to our knowledge that is unrelated to filamentation. In addition, these results, together with previous studies (21), suggest that Rme1 may mediate the impact of Ndt80 on filamentation, biofilm formation, and *UME6* expression. We consider each of these findings in turn.

Gene expression data provide a rationale for the variable fluconazole sensitivity phenotype of *ndt80*Δ/Δ mutants. Ndt80 activates expression of *CDR1*, a multidrug ABC transporter gene that can reduce intracellular azole accumulation (24). Our RNA-seq data set indicates that *CDR1* expression is reduced by 1.6-fold in the SC5314 *ndt80*Δ/Δ mutant, a relatively small change. Extensive analysis by Sellam et al. (11) has shown that Ndt80 activates many *ERG* genes as well. Our data set also indicates that nine ergosterol biosynthetic genes are downregulated significantly in the mutant, though most do not meet the log_2_ fold change threshold of −1. Again, the changes are small in view of how we think about cause-effect relationships. If we consider that *ndt80*Δ/Δ mutant fluconazole sensitivity arises from the cumulative effects of multiple small gene expression changes, it seems reasonable that the phenotype might vary among strains. In fact, genotype-phenotype variation often arises from an aggregate of small changes (28).

Why does the fluconazole sensitivity of the wild-type strains in our study vary? We have not found simple explanatory mutations in the Hirakawa data sets (23). However, we note that the relatively resistant strain P57055 expresses ~5-fold lower levels of *ERG3* than strain SC5314 in YPD medium, based on data of Xiong et al. (26). Erg3 activity yields toxic sterols in the presence of azoles due to its role in the alternative ergosterol pathway (29). A fivefold reduction in *ERG3* RNA levels has been shown to be sufficient to cause fluconazole resistance (30). The genetic variants in P57055 that cause reduced *ERG3* expression will be an interesting area for future study.

Ndt80 is known to govern filamentation and biofilm formation in the SC5314 strain background (9, 10, 13). However, many well-established regulators of filamentation and biofilm formation have more pronounced impact in some clinical isolates than others (18–22). We found that Ndt80 is required for normal filamentation in all clinical isolates tested. However, the phenotype is nuanced in two ways. First, it depends upon growth conditions, as does the native filamentation ability of each wild-type strain. Second, it affects the morphology of filamentous cells produced. We also found that Ndt80 is required for biofilm formation in all clinical isolates tested. A striking biofilm-associated phenotype—a patchy biofilm distribution—was observed in *ndt80*Δ/Δ mutants of all six clinical isolates. We do not know for certain the basis for this phenotype. We speculate that it reflects the heterogeneity of *ndt80*Δ/Δ mutant populations: perhaps a fraction of cells remain adherent and a fraction lose adherence. Overall, our results indicate that Ndt80 is required for filamentation and biofilm formation in all strains examined.

Which *NDT80* targets mediate its impact on filamentation and biofilm formation under our assay conditions? Candidates from our RNA-seq data included *UME6*, *WOR1*, and *RME1*. The regulatory regions of all three genes are bound by Ndt80, an indication that they are direct targets (13). Previous studies indicate that *UME6* expression is regulated by both Wor1 and Rme1 (19, 21). Therefore, we chose *UME6* as the most downstream effector that may exert Ndt80-dependent control over filamentation and biofilm formation. Suppression of the *ndt80*Δ/Δ filamentation and biofilm defects by *RBT5-UME6* was nearly complete, and RNA-seq data indicate that overexpression of *RBT5-UME6* RNA is ~2.5-fold, an increase that has little phenotypic or gene expression impact in wild-type SC5314. These results indicate that Ume6 is a major Ndt80 effector in the context of the filamentation and biofilm formation phenotypes.

Suppression of other *ndt80*Δ/Δ defects by *RBT5-UME6* was mixed. For example, only ~10% (69/777) of Ndt80-regulated genes were significantly affected by *RBT5-UME6*. This outcome may reflect that, in wild-type cells, Ume6 acts in part in a Ume6-Ndt80 complex. Most striking, though, was the combined impact of *ndt80*Δ/Δ and *RBT5-UME6* on fluconazole tolerance: *RBT5-UME6* did not suppress the increased *ndt80*Δ/Δ mutant drug sensitivity; instead, *RBT5-UME6* exacerbated it. We did not identify well-known genes that govern fluconazole vulnerability, such as *ERG* genes or efflux pump genes, that were impacted substantially in the *ndt80*Δ/Δ *RBT5-UME6* strain (Table S1). However, several upregulated genes specify iron-utilizing enzymes, an indication that the combination of *ndt80*Δ/Δ and *RBT5-UME6* may alter iron homeostasis. Ergosterol biosynthesis requires two heme-dependent enzymes (Erg11 and Erg5) and two iron-dependent enzymes (Erg25 and Erg3) (31). In *Saccharomyces cerevisiae*, iron deficiency impairs ergosterol accumulation (32). We suggest that increased expression of iron-utilizing enzyme genes in the *ndt80*Δ/Δ *RBT5-UME6* strain may limit the iron available for ergosterol biosynthetic enzymes and cause increased fluconazole susceptibility.

How may increased *UME6* expression alter *ndt80*Δ/Δ mutant phenotypes? Ume6 activates some genes through formation of an Ndt80-Ume6 complex (17). Absence of Ndt80 thus explains why increased *UME6* expression cannot fully suppress the *ndt80*Δ/Δ mutant phenotype. We hypothesize that *ndt80*Δ/Δ suppression by increased *UME6* expression results from activity of Efg1-Ume6 and Upc2-Ume6 complexes. Our RNA-seq data provide some support for this hypothesis. For example, *RBT5-UME6* increases expression of *HYR1*, *HWP1*, and *ERG251* in the *ndt80*Δ/Δ mutant (Table S1); Do et al. (17) found that Ume6 binding to these promoter regions is independent of Ndt80. *RBT5-UME6* does not increase expression of *ECE1* or *HGC1* in the *ndt80*Δ/Δ mutant (Table S1); Do et al. (17) found that Ume6 binding to these promoter regions depends upon Ndt80. The extreme fluconazole sensitivity of *ndt80*Δ/Δ *RBT5-UME6* strains may reflect activity of additional TF-Ume6 complexes not yet identified.

## MATERIALS AND METHODS

### Strains and culture conditions

Clinical isolates were obtained from Biodefense and Emerging Infections Research Resources Repository (BEI) resources National Institute of Allergy and Infectious Diseases, National Institutes of Health (NIH). *Candida albicans* strains SC5314, P76067, P57055, P87, P75063, and P78048 were used in this study (Table S2). Strains were stored long term in 15% glycerol solution at −80°C. Strains were grown out on YPD solid medium (2% peptone, 2% dextrose, 2% agar, and 1% yeast extract) at 30°C for 48 h before all experiments. The strains were cultured overnight in YPD liquid medium (2% peptone, 2% dextrose, and 1% yeast extract) at 30°C in a rotator drum.

### Transformations

Transformations of *Candida albicans* were performed in accordance with the transient CRISPR-Cas9 protocol (33). All primers and plasmids used in this study can be found in Table S2.

Deletion of *NDT80*: To delete *NDT80*, the *his1*Δ/Δ strains of each background listed above were transformed. One microgram of Cas9 DNA cassette, 1 µg of NDT80 sgRNA DNA cassette, 3 µg of ndt80::r1HIS1r1, and 1 µg of NAT1-5 sgRNA DNA cassette were used, with the Cas9 cassette amplified from plasmid pV1093 using primers CaCas9/F and CaCas9/R. The single guides were amplified using split-joint PCR with pV1093 and round 1 primers “NDT80 SNR52/R sg_,” “SNR52/F,” “NDT80 sgRNA/F sg_,” and “sgRNA/R,” round 2 using round 1 products, and round 3 utilizing “sgRNA/N” and “SNR52/N” to amplify the full single guide cassette. The *ndt80:r1HIS1r1* was generated in two parts: the first using pMH01 using “HIS CRIME adapF” and “NDT80 CRIME adapR” and pMH02 using “HIS CRIME adap R” and “NDT80 CRIME adapF.” Using the NAT1-5 sgRNA DNA cassette, it is possible to recycle the *NAT1* marker at the *his1*Δ::r3NAT1r3 locus due to Cas9-mediated double-stranded break to the repeat-flanked region (34). Using this technique, recombination is possible between the direct repeats of the marker, rendering the strain nourseothricin sensitive and leaving only a single copy of the repeat at the recycled locus. Transformants were selected on complete supplement mixture medium without histidine and were replica plated onto YPD plate + 400 µg/mL of nourseothricin (clonNAT, Gold Biotechnology) 48 h later to check nourseothricin sensitivity. These colonies were then streaked out for singles, gDNA isolated, and then further genotyped by PCR using primers: “NDT80 chk F,” “NDT80 chk int R,” and “Cd HIS1 Check int /R.”

Overexpression of *UME6* using the *RBT5* promoter: Overexpression strains were constructed by replacing 500 bp upstream of *UME6* with the selectable *NAT1* marker and the expressible *RBT5* promoter (27) with 80 bp of flanking homology to the up and downstream regions of the promoter being deleted using “RBT5 UME6/F” and “RBT5 UME6/R” and “UME6p sgRNA-2 /F” and “UME6p SNR52-2 /R,” which were amplified as previously described. Transformations were performed with 1 µg Cas9 DNA cassette, 1 µg *UME6p* sgRNA DNA cassette, and 3 µg of *UME6-RBT5 DC* cassette and then plated on YPD + 400 µg/mL of nourseothricin. Transformant genotypes were verified using the “UME6p check up /F,” “UME6p check int /R,” and “pNAT chk int R.”

### Spot plate assay

Cells were grown in 5 mL of liquid YPD rotating at 30°C for 18 h. Cells of each strain were then diluted in H_2_0 to an OD_600_ of 3.0 measured with a spectrophotometer. Fivefold dilutions were spotted (3 µL) using a multichannel pipette on YPD, with and without 50 µM of iron chelator bathophenanthrolinedisulfonate (BPS) (Sigma-Aldrich 11890) and/or fluconazole. Each spot plate was performed in at least three independent experiments conducted on different days.

### Biofilms

Strains were grown in 5 mL of liquid YPD rotating at 30°C for 18 h. One hundred microliters of liquid medium (RPMI or YPD) was prewarmed to 37°C in a 96-well plate (Greiner 96 wells Cat#655090). Wells were then inoculated to a final OD_600_ of 0.05 and incubated at 37°C for 90 min. Wells were then washed twice with 1× phosphate-buffered saline (PBS) to remove non-adherent cells, and 100 µL of fresh media was added to each well. Plates were then incubated at 37°C for 24 h at 60 rpm shaking. Supernatant was then removed, and biofilms were washed again with 1× PBS. Biofilms were fixed by adding 100 µL of 4% formaldehyde in 1× PBS and incubated at room temperature for 1 h. Biofilms were washed once more in 1× PBS and then stained overnight using 5.5 mg/mL of calcofluor white in 1× PBS. The biofilms were washed the next day and then clarified using a 50% thiodiethanol and 50% 1× PBS for 1 h. This was followed by a 1-h incubation with 100% thiodiethanol. Clarified biofilms were then imaged on Keyence fluorescence microscope using PlanFluor 20× 0.45/8.80–7.50 mm Ph1 objective with 2× digital zoom. Technical triplicate biofilms were imaged within the wells, and apical navigation images were taken to ensure even sampling. Each sample has been replicated in at least three independent biofilm assays conducted on different days. Please note that the rectangular boxes within each navigation view image are produced by the software, and we cannot readily remove them.

### Filamentation

Cells were grown in 5 mL of liquid YPD rotating at 30°C for 18 h. Pre-warmed 5 mL of YPD or RPMI was then inoculated to an OD_600_ of 0.5 from the overnight cultures and incubated for 4 h at 60 rpm. Cells were collected via centrifugation (2,800 rpm for 5 min) and fixed in 4% formaldehyde in 1× PBS for 15 min. The samples were then washed 1× in PBS and then stained using calcofluor white and proteinase K. Cells were imaged on a Keyence fluorescence microscope using PlanFluor 20× 0.45/8.80–7.50 mm Ph1 objective with 2× digital zoom. Images were taken in triplicate and analyzed on Fiji.

### RNA extraction and data analysis

Cells were grown in 5 mL of liquid YPD rotating at 30°C for 18 h. The next day, cells were inoculated to 25 mL of prewarmed RPMI-1640 media (Sigma-Aldrich, Inc., St. Louis, MO, USA) adjusted to pH 7.4 at an OD_600_ of 0.2. Cells were grown for 4 h at 225 rpm in a shaking incubator at 37°C, then harvested by vacuum filtration, and frozen at −80°C until RNA extraction. Three biological replicates were provided for RNA-seq experiments.

RNA extraction was performed according to previously published methods (18, 19). Cells were disrupted using Zirconia beads (Ambion, Fisher, Scientific, Waltham, MA, USA), and extraction was performed using a 25:24:1 phenol:chloroform:isoamyl alcohol method combined with a Qiagen RNeasy Mini Kit (Qiagen, Venlo, Netherlands). RNA-seq analysis and processing of raw fastq reads were performed by Novogene. Differential expression was assessed using DEseq2 (v 1.40.2) in R using alpha =0.05.

### Data interpretation

Interpretations and hypotheses were guided by the comprehensive information at the *Candida* Genome Database (25), FungiDB (35), and the Kyoto Encyclopedia of Genes and Genomes database (36). GO term enrichments were determined with FungiFun3 tools (37).

## Data Availability

Strains and plasmids are available upon request. The authors affirm that all data necessary for confirming the conclusions of the article are present within the article, figures, and supplemental materials. RNA-seq data have been deposited in the NCBI Gene Expression Omnibus with accession number GSE315271.

## SUPPLEMENTAL MATERIAL

Figure S1 msphere.00014-26-s0001.pdf Twenty-four-hour filamentation assays.

Figure S2 msphere.00014-26-s0002.pdf Fluconazole susceptibility assays.

Figure S3 msphere.00014-26-s0003.pdf Biofilm assays.

Table S1 msphere.00014-26-s0004.xlsx RNA-seq data.

Table S2 msphere.00014-26-s0005.xlsx Strains, oligos, and plasmids.

ASM does not own the copyrights to Supplemental Material that may be linked to, or accessed through, an article. The authors have granted ASM a non-exclusive, world-wide license to publish the Supplemental Material files. Please contact the corresponding author directly for reuse.
