# Supplementary material for: Impact of Candida albicans NDT80 and UME6 on biofilm formation and fluconazole susceptibility
Source: mSphere. 2026 Mar 27;11(4):e00014-26. doi: 10.1128/msphere.00014-26 (PMC13123709; doi:10.1128/msphere.00014-26)
Supplement: Figure S1 — Twenty-four-hour filamentation assays. [file msphere.00014-26-s0001.pdf]

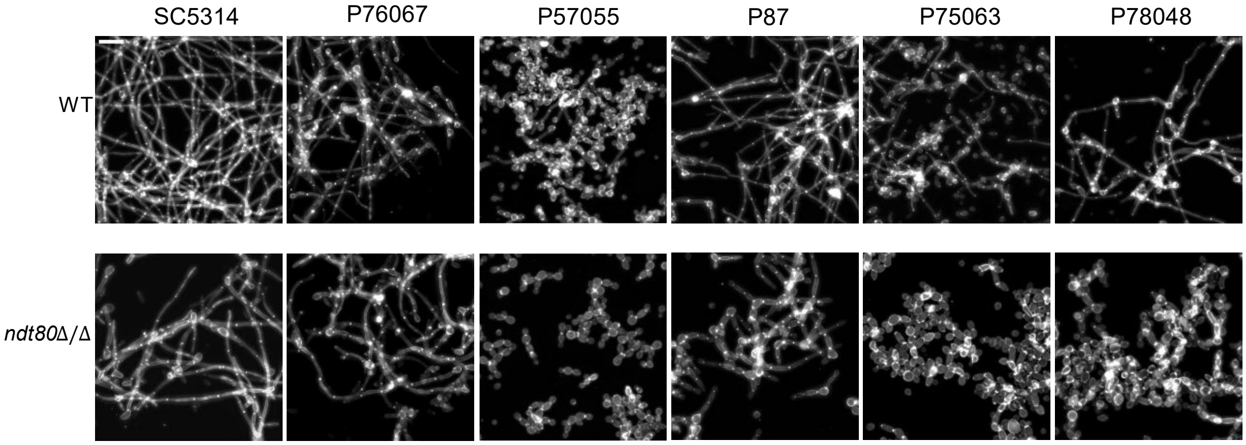

Supplementary Figure 1. 24-hour filamentation assays. Cells from YPD overnight cultures were inoculated into prewarmed RPMI and incubated with aeration for 24 hours at 37°C. Cells were then treated with proteinase K and stained with calcofluor-white. At least three fields of view were imaged, and representative images are shown. Each column represents a clinical isolate background, with the wild-type strain shown in the top row and the corresponding *ndt80*Δ/Δ mutant shown in the bottom row. The scale bar is 20 µm.
