# Supplementary material for: Impact of Candida albicans NDT80 and UME6 on biofilm formation and fluconazole susceptibility
Source: mSphere. 2026 Mar 27;11(4):e00014-26. doi: 10.1128/msphere.00014-26 (PMC13123709; doi:10.1128/msphere.00014-26)
Supplement: Figure S2 — Fluconazole susceptibility assays. [file msphere.00014-26-s0002.pdf]

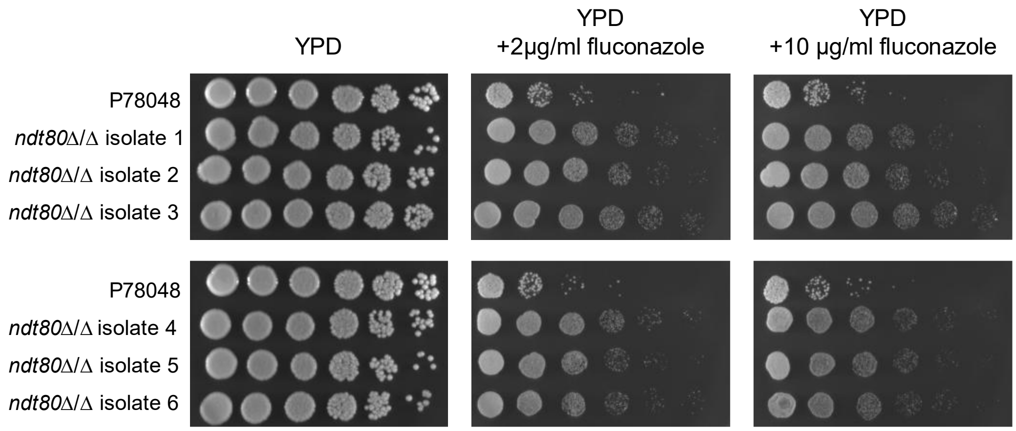

Supplementary Figure 2. Fluconazole susceptibility assays. Wild-type P78048 and 6 independent *ndt80*Δ/Δ mutants were assayed for fluconazole sensitivity. Strains were grown overnight in YPD at 30°C. Strains were then diluted to an OD600 ~3 and spotted on YPD, YPD + 2 µg/mL fluconazole, and YPD + 10 µg/mL fluconazole plates. Plates were then incubated at 30°C for 48 hours and imaged. Representative images are shown; each spot plate assay was performed in three independent experiments. Isolate 1 was the *ndt80*Δ/Δ mutant used in Figure 3.
