# Supplementary material for: Impact of Candida albicans NDT80 and UME6 on biofilm formation and fluconazole susceptibility
Source: mSphere. 2026 Mar 27;11(4):e00014-26. doi: 10.1128/msphere.00014-26 (PMC13123709; doi:10.1128/msphere.00014-26)
Supplement: Figure S3 — Biofilm assays. [file msphere.00014-26-s0003.pdf]

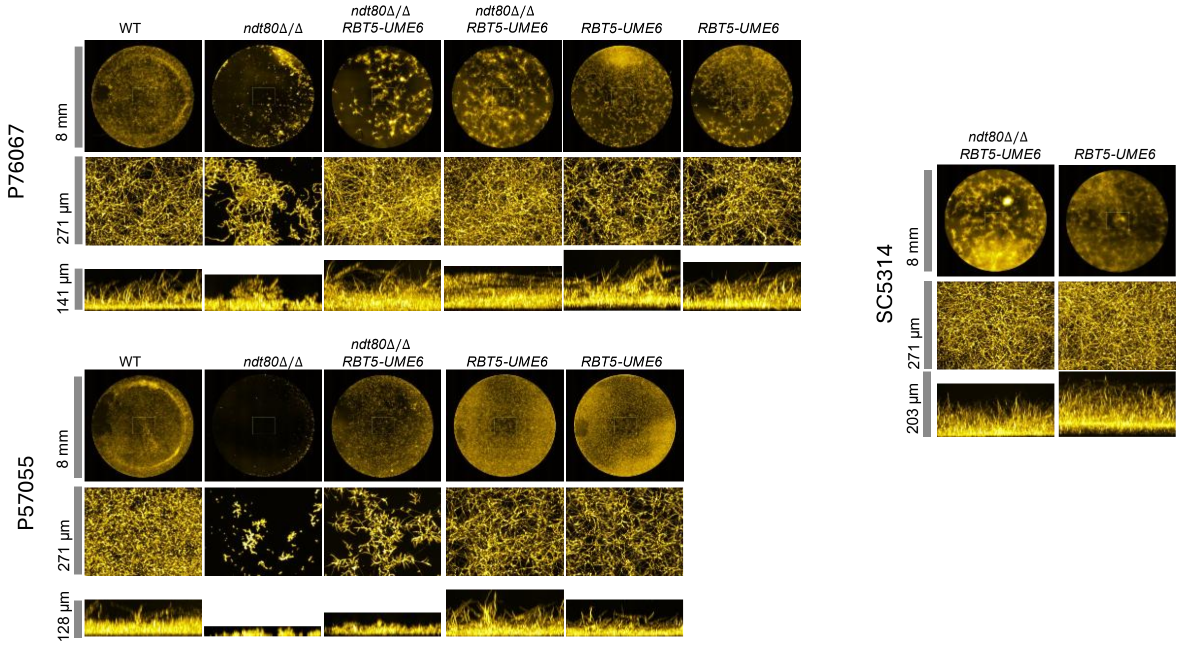

Supplementary Figure 3. Biofilm assays. Representative images of three independent samples used for calculations in Figure 5 are presented for strains P76067, P57055, and for two additional isolates of SC5314 *ndt80Δ/Δ RBT5-UME6* and *RBT5-UME6* strains. Cells were grown overnight in YPD + 50 µM BPS, then inoculated into prewarmed RPMI in 96 well plates and grown at 37°C for 24 hours. Biofilms were washed and stained with calcofluor white. Apical navigation views, apical projection views, and side projection views are shown. Scale bars are provided at the left of each row.
